# Hilar Biliary Cancer – are We Getting it Right?

**DOI:** 10.1155/1989/19817

**Published:** 1989

**Authors:** J. M. Little

**Affiliations:** Department of Surgery Westmead Hospital Westmead NSW 2145 Australia

## Abstract

Recent reviews of the management of hilar biliary cancer suggest that radical resection is the preferred treatment option. It seems true that most of the long term survivors are treated this way, but it is equally true that most patients receive palliative rather than curative treatment. Before we go too far in our advocacy of radical treatment, we need to develop methods for quantifying palliation because that is in truth what we usually achieve. A method of quantifying palliation is described. Surgeons are encouraged to use methods of this kind in order to allow valid comparison of the results of different methods of treatment.


*HPB Surgery* 1989, Vol. 1, pp. 93–96
Reprints available directly from the publisher
Photocopying permitted by license only

© 1989 Harwood Academic Publishers GmbH
Printed in Great Britain

### EDITORIAL COMMENT

### HILAR BILIARY CANCER - ARE WE GETTING IT RIGHT?

J.M. LITTLE*

*University of Sydney and Westmead Hospital, Westmead, NSW 2145, Australia*

*(Received 22 August 1988)*

Recent reviews of the management of hilar biliary cancer suggest that radical resection is the preferred treatment option. It seems true that most of the long term survivors are treated this way, but it is equally true that most patients receive palliative rather than curative treatment. Before we go too far in our advocacy of radical treatment, we need to develop methods for quantifying palliation because that is in truth what we usually achieve. A method of quantifying palliation is described. Surgeons are encouraged to use methods of this kind in order to allow valid comparison of the results of different methods of treatment.

KEY WORDS: Hilar biliary cancer, radical treatment, palliative treatment, quality of life

Those of us who went to Amsterdam to the Second Congress of the World Association of Hepatic, Pancreatic and Biliary Surgeons will have been impressed by many things — the superb organisation, the standard of the presentations, the high level of the discussions, the clear confirmation that our surgical speciality really exists. Those with an interest in bile duct cancer will have learnt much, but may have come away with a feeling of vague disquiet.

Certain messages were very clear. High bile duct cancer is fairly common in some parts of the world. Surgeons should adopt an "aggressive" approach to its treatment — and the word "aggressive" seemed to mean different things to different surgeons. At all events, resection of the tumour, with or without hepatic resection, emerged as the surgical ideal. Resection rates varied widely and without explanation. Launois reported a 50% resection rate, van der Heyde 16%. There seemed to be few cures to report, but survival times were felt to be better among those undergoing resection. Operative mortalities were "acceptable", and "good palliation" was reported among those who underwent resection but were not cured.

Some members of the audience left with the following ideas and impressions. A "significant number" of hilar bile duct cancers can be resected. "Radical" resection provides the best treatment. Selection of patients for radical resection depends more on operative assessment than on high technology staging preoperatively — in other words, exploratory surgery should be encouraged despite the inevitable morbidity in those patients with unresectable tumours and a correspondingly short life-span. There should be a general trend toward higher operation rates and more extensive resections. Those who are not joining this movement are not delivering an adequate and modern service, and their surgical manhood is at stake.

*Address for reprints: Professor J.M. Little, Department of Surgery, Westmead Hospital, Westmead, N.S.W. 2145, Australia.

But some serious anxieties persist, despite the eloquence of speakers and their persuasive arguments. No one really answered three central questions. What cases are not being referred to these special units that report such dazzling experience? What is happening to these unreferred cases, who treats them and how long do they survive? And what is the impact of surgery on the disease as a whole? Nor did anyone really report an attempt to measure the success of the palliation which is our fundamental aim, since "cures" are rare.

The recently reported experience of Bismuth's group[1] must sound a warning. Bismuth advocates the "radical, curative" approach to hilar cancer, but recognises with French lucidity that palliation is his usual achievement. He measures a "Comfort Index" for each patient treated by any means, which expresses the percentage of residual life experienced by the patient as good quality life. This approach is, of course, of fundamental value, although it is imprecise because no definition of good quality life (or "well being", as Bismuth puts it) is offered. What matters in the final analysis, however, is that Bismuth resects only 10% of the hilar cancers referred to his Unit, and that (if I interpret his figures correctly) only 20% of those undergoing resection will have a good life quality by the third postoperative year. In other words, only 2% of the whole group can be assumed to have benefitted from an aggressive resectional policy more than two years from the time of operation — and this assumption must be made without current information on the natural history of bile duct cancer, and in the context of the finding that involvement of the bile duct resection line may not significantly affect survival.[2]

Indeed, it is time to question the "radical" approach before it is too late, and we are forced to claim that no clinical trial can be justified. After all, Terblanche[3] has had good results for many years from intubation and radiotherapy. We really do not know whether the advent of radical surgery in the HPB field is a genuine advance, a relative disaster, a reinvention of the wheel or nothing more than an illusion. Radical operations in cancer surgery are not new. Massive ablation has been tried as treatment of carcinoma of the breast, stomach and lung in the not-so-distant past, and has been abandoned as each group has realised the inadequacy of a mechanical approach to a biological problem. We must ask ourselves whether there is something so very different about biliary cancer. Does it really stay confined to an operative field for so much longer than other cancers? After all, diagnosis is usually relatively late.

Whatever the answer to this last question, our specialty can certainly justify re-trying the radical approach. We have new diagnostic methods that are more precise than those available to the past generation. Our staging methods are correspondingly more precise. We have new anatomical and pathological information, and an improved understanding of the physiological disruptions suffered by the jaundiced patient. There are efficient anaesthetic and life support techniques, including methods of nutritional support. Our surgical techniques and technology are advancing. We have methods of maintaining life of reasonable quality after operation. We should, therefore, be able to do more surgically with a lower operative mortality and less long-term morbidity.

But if we are beguiled by our capacity for extended technical exercises, we may miss an essential point. The feasibility of an operation is not an indication for its performance. Resection rates for hilar cancer are seldom better than 20%, and 5 year cures are the exception. Whatever our stated aims may be, palliation is generally what we try to achieve. And no-one — except Bismuth[1] — seems to be trying to measure palliation.

Palliation is more than relief of symptoms. It also comprises an attempt to restore the best possible quality of life consistent with the stage of an incurable disease. An unconscious patient experiences no symptoms and no quality of life. Measurement of symptom severity has been used often enough. Various scales for pain measurement[4,5], for example, are well validated. Measurements of quality of life are harder. There are often culture-specific elements, but a number of scales have been developed that will probably cross national boundaries. This is not the place to review such scales, but the interested reader is referred to articles by Spitzer *et al.*[6], Little[7] and Clark and Fallowfield[8]. The development of better scales that might suit our specific needs will depend on surgeons cooperating with psychologists and sociologists — an unlikely collaboration, since surgeons regard social scientists as soft scientists, and social scientists dismiss surgeons as dehumanised non-scientists. These stereotypes need to be disregarded — some of my best friends are social scientists.

Despite the imperfections of the present quality of life scales, a method has been established for quantifying palliation[7]. It is based simply on regular personal follow-up, and an assessment of the chosen quality of life index at each visit. The index is plotted on a standard graph against time since intervention, and the area under the curve is calculated at set intervals and at death. The area represents the total number of time-quality-of-life units achieved for each patient. By comparing the actual achievement to the theoretically perfect figure, an efficiency index can also be calculated.

This method is being used to study the palliation achieved by surgical bypass and by percutaneous stent insertion. Patients are entered into the study without randomisation if malignancy is proven and they can be followed personally. They attend for assessment every second month. Patients are withdrawn from the study if they die before 6 months have passed since operation or stent insertion. The study is small, and there are only 15 patients available for assessment so far, 7 with stents, 8 having surgical bypass. Age, sex and survival times are comparable between the groups.

It is already evident that surgery reduces the patients' activity more markedly and for longer than stent insertion. By 6 months, cholangitis becomes a problem in the stented group, but not in those surgically bypassed. At 6 months, there is no statistical difference between the groups in quality of life. By 12 months, there is a clear advantage for the group having surgical bypass. It becomes important to see whether it is possible to find predictors of survival beyond 6 months.

High-powered basic research attracts money and prestige. It is generally only affordable in wealthy societies. Relevant clinical research is equally important, and can be done anywhere. To my mind, one of the strongest messages to emerge from the Amsterdam meeting was that many of the clinical questions of our time and our specialty are not answered and are not even being addressed. Research techniques for assessment of therapeutic results await development and refinement. Everyone can participate in these endeavours, which cost little and promise much. Everyone can define and begin to answer the questions posed by HPB diseases in their own countries.

On his deathbed, Isaac Newton said "I do not know what I may appear to the world; but to myself I seem to have been only like a boy playing on the seashore and diverting myself now and then finding a smoother pebble or a prettier shell than ordinary, whilst the great ocean of Truth lay all undiscovered before me." There is a great temptation to surgeons to play with the pebbles and shells of technical endeavour. It would be presumptuous to suggest that we can explore the ocean of truth, but it is timely to remember than an ocean of clinical observation and measurement lies before us. If the HPB Association achieved nothing but an agreement on ways to measure clinical results, it would have justified itself. If it could go further with joint studies that gave meaning to the experiences of surgeons from many countries, it would have achieved more than any comparable surgical society.

### *References*

1. Bismuth, H., Castaing, D., Traynor, O. (1988) Resection or palliation: priority of surgery in the treatment of hilar cancer. *World Journal of Surgery*, **12**, 39–47.
2. Gompertz, R.H., Benjamin, I.S., Blumgart, L.H. (1988) Hilar cholangiocarcinoma: what is curative resection? In *Abstracts of Papers presented at Second World Congress on Hepato-Pancreato-Biliary Surgery*, edited by H. Obertop, Bohn, Scholtema and Holkema, pp. 52 Utrecht/Antwerp.
3. Terblanche, J. (1979) Carcinoma of the proximal extrahepatic biliary tree — definitive and palliative treatment. *Surgery Annual*, **11**, 249–265.
4. Huskisson, E.C. (1983) Visual analogue scales. In *Pain Measurement and Assessment*, edited by R. Melzuck, pp. 33–37. New York: Raven Press.
5. Melzack, R. (1987) The short-form McGill Pain Questionnaire. *Pain*, **30**, 191–197.
6. Spitzer, W.O., Dobson, A.J., Hall, J. Chesterman, E., Levi, J., Shepherd, R. *et al.* (1981) Measuring the quality of life of cancer patients: a concise QL-index for use by physicians. *Journal of Chronic Diseases*, **34**, 585–597.
7. Little, J.M. (1987) A method of calculating the value of palliative care of cancer patients. *Australian and New Zealand Journal of Surgery*, **57**, 393–397.
8. Clark, A., Fallowfield, L.J. (1986) Quality of life measurements in patients with malignant disease. *Journal of the Royal Society of Medicine*, **79**, 165–169.

*Accepted by S. Bengmark on 22 August 1988.*

